# Continued anti-VEGF treatment does not prevent recurrences in eyes with stable neovascular age-related macular degeneration using a treat-and-extend regimen: a retrospective case series

**DOI:** 10.1038/s41433-021-01562-6

**Published:** 2021-05-03

**Authors:** Justus G. Garweg, Peter G. Traine, Richard A. Garweg, Juliana Wons, Christin Gerhardt, Isabel B. Pfister

**Affiliations:** 1 Swiss Eye Institute, Rotkreuz, and Berner Augenklinik am Lindenhofspital, Bern, Switzerland; 2 Department of Ophthalmology, Inselspital, Bern University Hospital, University of Bern, Bern, Switzerland; 3 University of Bern, Bern, Switzerland; 4 Private Practice, Steffisburg, Switzerland

**Keywords:** Macular degeneration, Retinal diseases

## Abstract

**Background:**

The continuation of anti-vascular endothelial growth factor (anti-VEGF) treatment after achieving stability in patients with neovascular age-related macular degeneration has generally been advocated. In our own patients, we thought to assess whether continued anti-VEGF treatment is capable of preventing recurrences.

**Methods:**

In this retrospective observational case series, patients with stable disease either opted to continue treatment every 12–14 weeks (Group 1) or stopped treatment with subsequent follow-up visits every 8–12 weeks (Group 2).

**Results:**

Of the 103 eyes of 103 patients achieving stability, 49 eyes continued treatment (Group 1), whereas treatment was stopped in 54 eyes undergoing regular follow-up (Group 2). Recurrent disease was observed in 21 (42.9%) and 33 (61.1%) cases in Group 1 and Group 2, respectively (*p* = 0.08). Time between achieving stable disease and recurrence was comparable between Group 1 and Group 2 (11.1 ± 8.2 months vs. 9.2 ± 6.7 months; *p* = 0.43). The number of visits between achieving stability and disease recurrence was similar, but not the number of injections (3.5 ± 2.0 vs. 0.2 ± 0.4; *p* < 0.001).

**Conclusions:**

Continuing anti-VEGF therapy after achieving functional and morphological stability every 12–14 weeks does not prevent recurrences. Patients deserve to be informed of a potential lifetime risk of recurrences, even under continued therapy.

## Introduction

Neovascular age-related macular degeneration (nAMD) causes a gradual loss of vision if left untreated [1–3]. Most patients achieve remarkable, although incomplete, functional restoration within a few months after initiating treatment [4, 5]. The potential for visual gain is primarily achieved by the end of the loading phase, with generally stable visual function. Further treatment aims to maintain visual function over the following years [6–8]. At this stage, the condition meets the criteria for chronic disease, in which patients and their families need to learn and come to terms with, making long-term medical management demanding. As patients become older and more fragile during long-term follow-up with concurrent substantial chronic health conditions, regular visits to their treating ophthalmologist become a challenge. At a certain stage, patients wish to pause treatment due to their perception of treatment futility.

Pro re nata (PRN), or as needed treatment, showed that vision loss occurs despite monthly follow-up and immediate resumption of therapy upon reactivation after a pause in treatment [9, 10]. The CATT study reported a lesion growth of 30–50% in the second year using the PRN protocol, resulting in the permanent vision loss after 2–4 years [11]. Consequently, this protocol has been replaced by a treat-and-extend (T&E) regimen in most centres. The T&E protocol allows treatment intervals to be extended up to a maximum of 14 weeks, according to the individual eye’s needs [12]. An extension beyond 16 weeks has not been generally advocated [13] because of an increased risk of reactivation and preventable vision loss. Two recent studies showed that recurrence with functional impact should be expected in 13–79% of cases after 1–5 years [14, 15]. Therefore, this purported exit strategy exposes the eye to the risk of recurrences and associated permanent vision loss. In contrast, ongoing treatment every 12–14 weeks might prevent recurrences and corresponding vision loss, although this has not yet been demonstrated. Evidence-based practice is, therefore, limited [14, 15], and any conclusions based thereon are potentially premature [16]. The present study intended to close this gap and assessed the recurrence incidences in eyes under continuous treatment (every 12–14 weeks), and those with cessation of treatment following an exit strategy with follow-ups every 8–12 weeks for at least 12 months.

## Methods

This single-centre, retrospective, observational, consecutive case series included patients, who were being treated for newly diagnosed nAMD with an approved intravitreal anti-vascular endothelial growth factor (anti-VEGF) agent (ranibizumab or aflibercept), using a T&E regimen. The study was performed at the Berner Augenklinik am Lindenhofspital, Bern, Switzerland between December 2012 and December 2018. The total follow-up time was a minimum of 2 years. The minimum follow-up for patients after treatment cessation was 24 months or 12 months after recurrence. The initial diagnosis was confirmed using fluorescein angiography and optical coherence tomography (OCT). The T&E regimen consisted of three loading injections at monthly intervals without intercurrent visits. Treatment intervals were extended by 2 weeks until a maximal interval of 14 weeks if stable disease was present. For the purpose of this study, stable disease was defined as the absence of intraretinal fluid, absent or stable subretinal fluid and/or pigment epithelial detachment compared to the last visit, whereas functional stability was not required. The T&E regimen planned a maximal tolerance of 15% of scheduled appointments, with a minimum of six injections in the first year. A total of 317 patients had to be excluded for the following reasons: patients were not treated adhering to the T&E protocol, did not achieve stability, did not comply with scheduled appointments (i.e., those not attending more than one clinical visit or injection per year) and treatment was terminated due to futility. Reasons for futility included macular scar or atrophy, preventing a visual gain upon treatment or visual function not likely to result in further vision loss after treatment cessation (best-corrected visual acuity (BCVA) ≤ 20/400 corresponding to 25 Early Treatment of Diabetic Retinopathy Study (ETDRS) letters).

After reaching stability, intravitreal injection was administered every 12–14 weeks or treatment was terminated with regular follow-up visits planned every 2–3 months. After receiving information from their treating physician about the potential risks and benefits of continuing treatment or its cessation, patients could decide themselves whether or not to continue with the intravitreal injections once they had reached stability. Eyes that continued intravitreal injection therapy were classified as Group 1 (continuous treatment every 12–14 weeks), whereas eyes in which intravitreal anti-VEGF therapy was interupted after achieving stability were defined as Group 2 (exit). Disease reactivation was assumed in the case of new intraretinal, or changes in subretinal or sub-pigment epithelial fluid and haemorrhages in comparison to the previous visit.

The primary outcome measure was the proportion of patients with recurrent nAMD activity after achieving stable disease without (Group 1) or after pausing treatment (Group 2). Secondary outcome measures were time to disease recurrence, the number of visits and injections between the time of stabilization and disease reactivation or 12 months thereafter, as well as BCVA values.

Visual acuity testing (Snellen BCVA) and OCT (central horizontal line scan of 6 mm using a Spectralis^®^, Heidelberg Engineering, Heidelberg, Germany) were recorded at each clinical visit to monitor disease activity. BCVA, central retinal thickness (CRT) and functionally relevant anatomic findings, such as intraretinal or subretinal fluid and presence of new haemorrhages, were retrospectively retrieved from the patients’ electronic records. BCVA values were converted to the corresponding ETDRS letter score [17], with a BCVA of 1.0 equivalent to 85 ETDRS.

Ethics approval was obtained from the Cantonal Ethics Committee in Bern (KEK 2019-01914). All patients provided informed consent for the use of their coded data. The study was conducted in compliance with the tenets of the Declaration of Helsinki.

## Statistical analysis

To ensure that our statistical tests would have adequate power, before starting data collection, we calculated the sample size necessary to detect an effect. We applied the formula for sample size calculation for testing a hypothesis with qualitative data [18]. Based on previous findings, we wanted to uncover differences in visual acuity of ±10 ETDRS letters, with a standard deviation (SD) of ~15 letters. Applying this to the formula above, at 5% type I error, results revealed a sample of 35 eyes per group to be enough to uncover an effect at 80% power.

The Shapiro–Wilk test indicated that the data were not normally distributed; therefore, nonparametric tests were applied. The chi-square test and Mann–Whitney *U* test were applied to test for intergroup differences. Data are presented as mean ± SD. All statistical analyses were performed using the SPSS software package V.23 (SPSS, Inc., Chicago, Illinois, USA). The level of significance was set at *p* < 0.05.

## Results

Out of 420 patients, a total of 103 eyes (103 patients) fulfilled the inclusion criteria for the study. A larger part of 171 patients never achieved disease stability; 104 patients did not follow the T&E protocol; 35 patients had <2 years of follow-up and in 7 patients, treatment was stopped when very low vision (≤20/400) and a lack of visual potential were observed. The remaining study sample achieved stable disease under ≥12-week injection intervals after an average of 22.7 ± 9.8 weeks and 11.7 ± 5.0 injections following a T&E protocol, according to the above-mentioned eligibility criteria. Forty-nine patients continued intravitreal treatment every 12–14 weeks (Group 1), whereas 54 patients opted for treatment cessation after achieving stability criteria (Group 2). Baseline characteristics, including age, gender and lens status, were comparable for both groups (Table 1).

**Table 1:** Epidemiological data, time intervals, visits and intravitreal injections during the study period.

	Group 1 (no exit; *n* = 49)	Group 2 (exit; *n* = 54)	*P* value^b^
Epidemiological data			
Age (years: mea ± SD, min–max)	77.5 ± 7.8, 54.9–89.4	78.0 ± 8.6, 58.3–99.8	0.87
Gender (% females)	67.3	55.6	0.23
Pseudophakia (%)	44.9	46.3	1.0
Total follow-up time (years: mean ± SD, min–max)	3.4 ± 1.0, 2.0–6.0	4.0 ± 1.1, 1.7–6.2	0.005
Recurrence, *n* (%)	21 (42.9)	33 (61.1)	0.08
Time intervals (months: mean ± SD, min–max)			
Treatment initiation until treatment stabilization^a^	23.2 ± 9.4, 10.1–47.9	22.3 ± 10.1, 10.3–59.4	0.51
Treatment stabilization (Group 1)/treatment cessation (Group 2) until reactivation	11.1 ± 8.2, 2.8–33.2	9.2 ± 6.7, 1.6–31.7	0.43
Visits (*n*, mean ± SD, min–max)			
Treatment initiation until treatment stabilization	8.5 ± 3.6, 4–16	7.4 ± 3.5, 4–22	0.09
Treatment stabilization/ treatment cessation until reactivation	4.2 ± 3.0, 1–13	3.9 ± 2.4, 1–12	0.87
Between reactivation and 12 months thereafter	6.2 ± 1.5, 4–9	6.0 ± 1.6, 4–9	0.59
Intravitreal anti-VEGF injections (*n*, mean ± SD, min–max)			
Treatment initiation until treatment stabilization	12.4 ± 5.1, 6–31	11.0 ± 4.8, 5–27	0.07
Treatment stabilization/ treatment cessation until reactivation	3.5 ± 2.0, 1–8	0.2 ± 0.4, 0–1	0.0005
Between reactivation and 12 months thereafter	5.1 ± 1.7, 2–9	4.5 ± 1.9, 1–8	0.27

^a^Treatment stabilization: no disease activity after two ≥12-week treatment intervals without clinical or OCT evidence-based reactivation.

^b^Chi-square test and Mann–Whitney *U* test were applied to test for intergroup differences.

**Fig. 1:**
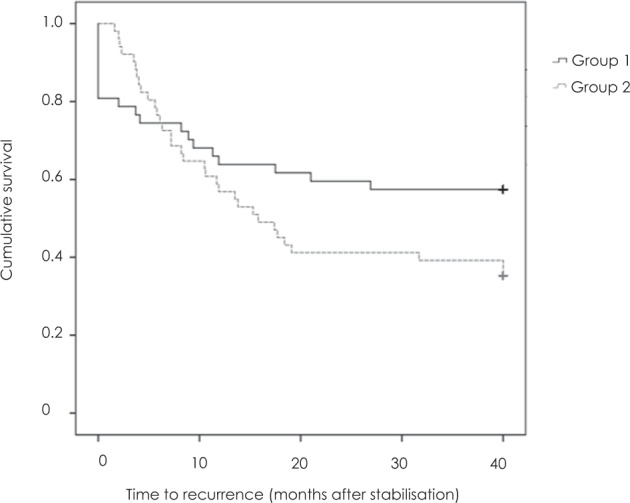
Time between stabilisation and reactivation of macular neovascularisation. Kaplan–Meier estimate of time from achieving stable disease (two ≥12-week intervals without evidence of reactivation) to recurrence of neovascular age-related macular degeneration (*p* = 0.24).

Recurrence was observed in 21 of 49 eyes (42.9%) in Group 1, with reactivation occurring after a mean of 11.1 ± 8.2 (2.8–33.2) months after achieving stable disease. In Group 2, 33 of 54 eyes (61.1%; *p* = 0.08) experienced recurrence, which was diagnosed after a mean of 9.2 ± 6.7 (1.6–31.7, *p* = 0.43) months after treatment cessation (Table 1 and Fig. 1).

**Fig. 2:**
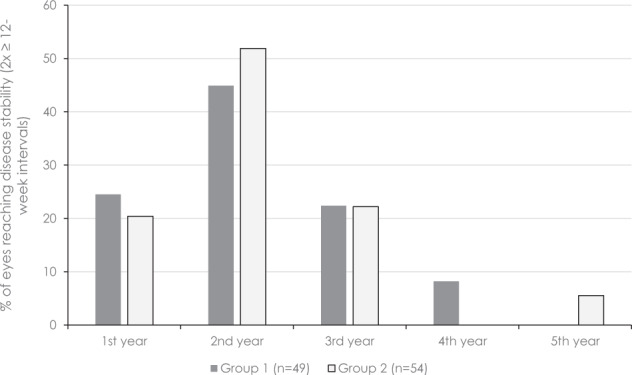
Time between treatment initiation and achieving stability in macular neovascularisation. Time interval between treatment initiation to achieving stability (two ≥12-week intervals without clinical or OCT evidence-based reactivation; *p* = 0.14).

Similar time intervals were found in both groups from treatment initiation until achieving stability and time to reactivation (Table 1). No difference in the number of visits was found between the groups, but as expected, there was a significant difference in the number of injections. Per definition, the number of injections between treatment cessation and reactivation approached zero in Group 2. As expected, the time from treatment initiation to achieving stability varied consistently within, however, not between the two groups (23.2 ± 9.4 (10.1–47.9) months vs. 22.3 ± 10.1 (10.3–59.4) months; *p* = 0.51; Fig. 2).

**Fig. 3:**
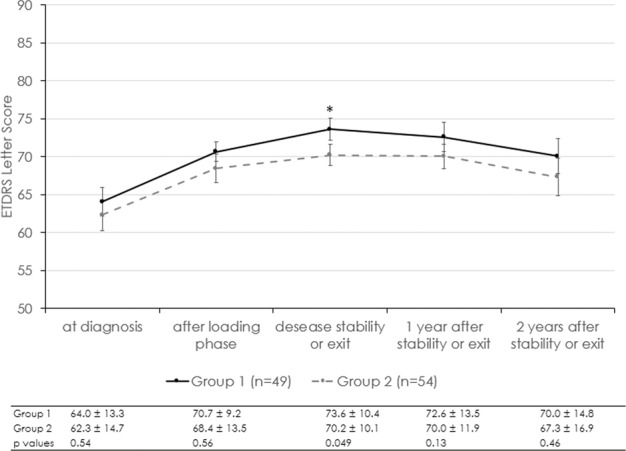
Evolution of visual acuity. Evolution of best-corrected visual acuity from diagnosis and after achieving stability over 2 years.

Visual acuity was also similar between the two groups (Fig. 3).

## Discussion

Reactivation of nAMD is a common finding in patients treated with currently available anti-VEGF therapies and can affect eyes after a long-term stable phase independent of the treatment protocol [16]. Since treatment intensity in T&E regimens is directed towards the lower end of morphologically detectable disease activity, this occurs in any biologically active, partially fibrotic lesion. Morphological parameters, namely the dynamics of intraretinal and/or subretinal and sub-pigment epithelial fluid, are criteria to adopt retreatment intervals to the minimal individual patient’s needs [12]. However, OCT-based disease activity criteria lag behind reperfusion and leakage from a pre-existing neovascular lesion [19, 20]. Tissue VEGF concentrations will increase over time until achieving sufficient levels to induce detectable reactivation. OCT angiography-driven retreatment decisions may have the future potential to close this gap [21, 22]. Longer acting anti-VEGF drugs and drug delivery systems will further add in extending the time to recurrence, whereas there is no treatment on the horizon aiming at treating the underlying disease pathophysiology, allowing a curative approach [13, 23–26].

Currently, a good early response to anti-VEGF treatment [27], treatment duration and the duration of the stability phase before reaching exit criteria [15] have been identified as potential predictors for long-term success and risk of reactivation. In this study, 25% of examined eyes achieved stability and met their exit criteria after two injection intervals of 12–14 weeks, whereas a higher proportion of eyes might have been stable under an injection interval of 10–12 weeks or shorter [28]. In line with this assumption, Nguyen and colleagues reported a recurrence rate of 41% after treatment cessation, following a single 12-week interval [29]. Extending the maximal interval to 16 weeks for 1 year before treatment cessation, in contrast, reduced the number of eyes meeting the treatment stop criteria to 13% [15], whereas the remaining eyes likely lay at the threshold of visible disease activity, which became evident during the longer follow-up interval prior to reaching the exit criteria. These observations led to the conclusion that a longer disease-free interval would allow a reduction in the risk of recurrence and, therefore, represent a better selection of eyes for treatment cessation [15]. In our opinion, this remains rather speculative. These results demonstrate that the longer a preventive T&E treatment is performed without complete VEGF suppression, the higher the chance of disease reactivation before treatment cessation, resulting in lower recurrence rates in the remaining eyes. In line with this assumption, five eyes (9.8%) under continuous treatment in the present study experienced a recurrence within 6 months of achieving stable disease, which is comparable to the study conducted by Nguyen and colleagues [27]. Finally, we observed the time from achieving stable disease to recurrence to be generally identical for patients under continuous treatment compared to that in patients after treatment cessation. In contrast to previous studies [14–16], our data demonstrate that continuous treatment does not prevent recurrence. This points to the continued lack of critical parameters predicting recurrence.

Despite a total treatment duration of 4.5 years and disease stability of 62–74 weeks, reactivation within a mean of 37 weeks (2.3 times the last treatment interval before treatment cessation) was reported in Arendt’s study. This is well in line with our findings, in 13% of eyes does not argue in favour of the strong impact of total treatment duration or interval between stability and treatment cessation [15]. Yet, treatment duration has also been identified in a second retrospective analysis [29]. In this registry-based study, an indirect correlation between treatment duration and visual acuity at treatment suspension appears to exist. Eyes with longer anti-VEGF treatment duration and eyes with a very low visual acuity (<35 letters; <0.1 Snellen visual acuity) were reported to have a low recurrence rate. However, there may be a wide overlap between these two groups. Eyes with very low vision [14], which were excluded in our study (*n* = 7), likely terminated and did not resume treatment because of a lack of visual potential, which does not prove lesion stability. Thus, early response to treatment remains the only possible factor predicting recurrences.

A major limitation of this study is its retrospective design, although strictly following a T&E protocol. A bias resulting from case selection can widely be excluded since only treatment-naive eyes with a confirmed diagnosis and intravitreal treatment initiated within the index period were included. Based on limited published evidence, patients were informed about the potential risks and benefits of treatment continuation or cessation before opting to continue or pause the treatment. It may be assumed that patients with a lower visual acuity tended to decide on treatment cessation, whereas better vision resulted in the decision for treatment continuation. Based on a mean BCVA of 70 letters vs. 73 letters and a 3.4 letter difference, this is unlikely to have had a major impact on the treatment decision. Moreover, leaving the decision regarding the treatment strategy to the informed patients may harbour potential bias. Half of the patients decided for treatment cessation and half decided against treatment cessation. Most likely, this decision was based on the previous treatment experience. This bias, whatever its dimension may have been, does not change the key message that disease reactivation is unescapably arising in the course of disease, be it treated or not (at least under a T&E protocol). Strong adherence to the treatment protocol represents a potential strength of the study; however, based thereon, 70% of patients did not fulfil the inclusion criteria, because the vast majority did not reach stability (*n* = 171) or did not follow the scheduled visits requested for the T&E protocol. Whereas, 8% of patients (*n* = 33) did not reach the minimum follow-up after achieving stability. The relatively low resulting number of included patients argues against drawing any conclusions beyond that reactivation is based in the nature of the disease. Recurrence is seemingly unpreventable with available therapeutics, except possibly by permanent VEGF activity suppression, at the expense of potential long-term side effects, such as a more rapid geographic atrophy growth. This will turn out to be the case with the introduction of anti-VEGF drug delivery systems, which are currently under investigation.

In conclusion, our results add to existing evidence that recurrence cannot be prevented by continuing anti-VEGF treatment after achieving stable disease and extending the treatment interval to 12 or more weeks in a relevant portion of patients, which lies in the nature of the underlying disease and its biological basis. The application of newer tools and strategies, namely OCT angiography, to determine disease activity [30], may help to reduce the risk of vision loss until new therapeutic targets have been identified that enable a pathophysiological-based therapy.

### Summary

#### What was known before

- Much is known about the treatment of AMD with anti-VEGF drugs. Preventing recurrences in eyes with stable nAMD is important. Nothing is known about the best time to pause treatment after stability has been achieved.

#### What this study adds

- Continuing anti-VEGF therapy after achieving functional and morphological stability every 12–14 weeks does reduce, but not prevent recurrences.

## References

[1] Rosenfeld PJ, Brown DM, Heier JS, Boyer DS, Kaiser PK, Chung CY (2006). MARINA Study Group. Ranibizumab for neovascular age-related macular degeneration. N. Engl J Med.

[2] Brown DM, Kaiser PK, Michels M, Soubrane G, Heier JS, Kim RY (2006). ANCHOR Study Group. Ranibizumab versus verteporfin for neovascular age-related macular degeneration. N. Engl J Med.

[3] Abraham P, Yue H, Wilson L (2010). Randomized, double-masked, sham-controlled trial of ranibizumab for neovascular age-related macular degeneration: PIER study year 2. Am J Ophthalmol.

[4] Heier JS, Brown DM, Chong V, Korobelnik J, Kaiser PK, Nguyen QD (2013). VIEW 1 and VIEW 2 Study Groups. Intravitreal aflibercept (VEGF trap-eye) in wet age-related macular degeneration. Ophthalmology.

[5] Waldstein SM, Simader C, Staurenghi G, Chong NV, Mitchell P, Jaffe GJ (2016). Morphology and visual acuity in aflibercept and ranibizumab therapy for neovascular age-related macular degeneration in the VIEW Trials. Ophthalmology.

[6] Ho AC, Busbee BG, Regillo CD, Wieland MR, Van Everen SA, Li Z (2014). Twenty-four-month efficacy and safety of 0.5 mg or 2.0 mg ranibizumab in patients with subfoveal neovascular age-related macular degeneration. Ophthalmology.

[7] Rofagha S, Bhisitkul RB, Boyer DS, Sadda SR, Zhang K, SEVEN-UP Study Group. (2013). Seven-year outcomes in ranibizumab-treated patients in ANCHOR, MARINA, and HORIZON: a multicenter cohort study (SEVEN-UP). Ophthalmology.

[8] Kaiser PK, Singer M, Tolentino M, Vitti R, Erickson K, Saroj N (2017). Long-term safety and visual outcome of intravitreal aflibercept in neovascular age-related macular degeneration: VIEW 1 Extension Study. Ophthalmol Retin.

[9] Richard G, Mones J, Wolf S, Korobelnik JF, Guymer R, Goldstein M (2015). Scheduled versus pro re nata dosing in the VIEW trials. Ophthalmology.

[10] Berg K, Hadzalic E, Gjertsen I, Forsaa V, Berger LH, Kinge B (2016). Ranibizumab or bevacizumab for neovascular age-related macular degeneration according to the Lucentis Compared to Avastin Study treat-and extend protocol: two-year results. Ophthalmology.

[11] Martin DF, Maguire MG, Fine SL, Ying G, Jaffe GJ, Grunwald, CATT Research Group (2012). Ranibizumab and bevacizumab for treatment of neovascular age-related macular degeneration: two-year results. Ophthalmology.

[12] Amoaku WM, Chakravarthy U, Gale R, Gavin M, Ghanchi F, Gibson J (2015). Defining response to anti-VEGF therapies in neovascular AMD. Eye.

[13] Mehta H, Tufail A, Daien V, Lee AY, Nguyen V, Ozturk M (2018). Real-world outcomes in patients with neovascular age-related macular degeneration treated with intravitreal vascular endothelial growth factor inhibitors. Prog Retin Eye Res.

[14] Nguyen V, Vaze A, Fraser-Bell S, Arnold J, Essex RW, Barthelmes D (2019). Fight Retinal Blindness! Study Group. Outcomes of suspending VEGF inhibitors for neovascular age-related macular degeneration when lesions have been inactive for 3 months. Ophthalmol Retin.

[15] Arendt P, Yu S, Munk MR, Ebneter A, Wolf S, Zinkernagel MS (2019). Exit strategy in a treat and extend regime for exudative age related macular degeneration. Retina.

[16] Khurana RN (2019). Long-term management of neovascular age-related macular degeneration: to suspend or not to suspend?. Ophthalmol Retin.

[17] Gregori NZ, Feuer W, Rosenfeld PJ (2010). Novel method for analyzing snellen visual acuity measurements. Retina.

[18] Charan J, Biswas T (2013). How to calculate sample size for different study designs in medical research?. Indian J Psychol Med.

[19] Lowry EA, Nguyen V, Daien V, Barthelmes D, Gillies MC (2018). Outcomes in neovascular age-related macular degeneration when neovascular lesion activity is uncertain: observational study. Ophthalmol Retin.

[20] Yang J, Zhang Q, Motulsky EH, Thulliez M, Shi Y, Lyu C (2019). Two-year risk of exudation in eyes with non-exudative AMD and subclinical neovascularization detected with swept source OCT angiography. Am J Ophthalmol.

[21] Zhang Q, Chen CL, Chu Z, Zheng F, Miller A, Roisman L (2017). Automated quantitation of choroidal neovascularization: A comparison study between spectral-domain and swept-source OCT angiograms. Investig Ophthalmol Vis Sci.

[22] Miller AR, Roisman L, Zhang Q, Zheng F, de Olivera Dias JR, Yehoshua Z (2017). Comparison between spectral-domain and swept-source optical coherence tomography angiographic imaging of choroidal neovascularization. Investig Ophthalmol Vis Sci.

[23] Ashraf M, Souka A, Adelman RA (2018). Age-related macular degeneration: using morphological predictors to modify current treatment protocols. Acta Ophthalmol.

[24] Jones MK, Lu B, Girman S, Wang S (2017). Cell-based therapeutic strategies for replacement and preservation in retinal degenerative diseases. Prog Retin Eye Res.

[25] Kroeger H, Chiang WC, Felden J, Nguyen A, Lin JH (2019). ER stress and unfolded protein response in ocular health and disease. FEBS J.

[26] Gutierrez MA, Davis SS, Rosko A, Nguyen SM, Mitchell KP, Mateen S (2016). A novel AhR ligand, 2AI, protects the retina from environmental stress. Sci Rep.

[27] Nguyen V, Daien V, Guymer R, Young S, Hunyor A, Fraser-Bell S (2019). Fight Retinal Blindness! Study Group. Projection of long-term visual acuity outcomes based on initial treatment response in neovascular age-related macular degeneration. Ophthalmology.

[28] Essex RW, Nguyen V, Walton R, Arnold JJ, McAllister IL, Guymer RH (2016). Fight Retinal Blindness Study Group. Treatment patterns and visual outcomes during the maintenance phase of treat-and-extend therapy for age-related macular degeneration. Ophthalmology.

[29] Nguyen CL, Gillies MC, Nguyen V, Daien V, Cohn A, Banerjee G (2019). Fight Retinal Blindness! Study Group. Characterization of poor visual outcomes of neovascular age-related macular degeneration treated with anti-vascular endothelial growth factor agents. Ophthalmology.

[30] Schmidt-Erfurth U, Waldstein SM (2016). A paradigm shift in imaging biomarkers in neovascular age-related macular degeneration. Prog Retin Eye Res.

